# Technical note: Correlation between TQA data trends and TomoHD functional status

**DOI:** 10.1120/jacmp.v15i2.4548

**Published:** 2014-03-06

**Authors:** Hervé H.F. Choi, Joan P.Y. Ho, Bin Yang, Kin Yin Cheung, Siu Ki Yu

**Affiliations:** ^1^ Medical Physics and Research Department Hong Kong Sanatorium & Hospital Happy Valley Hong Kong

**Keywords:** accelerators in radiation therapy, quality assurance for radiation therapy equipment, magnetrons

## Abstract

TomoTherapy Quality Assurance (TQA) is a software package developed to monitor certain aspects of machine performance. In this study, the TQA quantities or data trends most effective in monitoring energy drifts and magnetron stability were determined respectively. This retrospective study used data collected from three TomoHD units. The TQA modules investigated were Step‐Wedge Helical, Step‐Wedge Static, and Basic Dosimetry. First, the TQA quantities correlated with energy changes (|r|>0.85, where *r* is the Pearson's correlation coefficient) were found. The corresponding sensitivities to percentage depth dose (PDD) ratio changes were then calculated and compared. Second, the pulse‐by‐pulse dose stability was compared before and after each magnetron replacement using a nonparametric comparison test (Welch's t‐test), and the raw dose profiles were surveyed. In this study, exit detector flatness obtained in Basic Dosimetry was shown to be the most sensitive (r=0.945) to energy changes, followed by the energy differences in Step‐Wedge Static (r=0.942) and Step‐Wedge Helical (r=0.898). The three quantities could detect a PDD ratio change of $5.1\times 10^{-4},5.4\times 10^{-4}$, and $7.1\times 10^{-4}$, respectively. Pulse‐by‐Pulse Dose1 from Basic Dosimetry over a one‐week period before and after a magnetron replacement showed a significant difference (p<0.05) in only three of the nine instances. On the other hand, a raw output profile free from discontinuities, frequent dropped pulses and abnormal spikes was found to indicate that the magnetron would continue to function normally for a week 89% of the time.

PACS numbers: 87.56.bd, 87.56.Fc, 84.40.Fe

## I. INTRODUCTION

TomoTherapy Quality Assurance (TQA) is a TomoTherapy (Accuray, Sunnyvale, CA) software package used for monitoring the functional status of a TomoTherapy unit. The data are first collected by the onboard detector, and then uploaded and stored online. Data trends can subsequently be recalled and analyzed later.^(1)^ Among the vast data collected in various TQA modules, some provide similar information about the functional status of the machine.

Four TQA modules — Basic Dosimetry, System Monitor, Step‐Wedge Static, and Step‐Wedge Helical^1^, — are run on the regular basis for each of the three TomoHD units at our center. Basic Dosimetry assesses the beam output based on rotational variation, System Monitor provides an overview of the parameters essential for the functioning of the machine, while the two Step‐Wedge modules provide further information of the beam using the attenuation of an aluminum step wedge. Among the four, System Monitor measurements are not calibrated or traceable, and are not used in this note. In this technical note, we explore if there is a TQA quantity that can most effectively track changes in the beam energy, as well as a TQA data trend that correlates most strongly with magnetron failures.

## II. MATERIALS AND METHODS

The data used in this retrospective study are collected from three TomoHD units at our center over a minimum period of nine months. The units are not equipped with a dose servo, which have been demonstrated to exhibit a rotational output variation of ±2%.^(2)^ The three machines have undergone, in total, nine magnetron replacements due to magnetron failure; further, one of the three TomoHD units has undergone two energy adjustments. To monitor the performance of TomoHD units, the module Basic Dosimetry has been run at least once a day, whereas the modules Step‐Wedge Static and Step‐Wedge Helical are run once a week.

### A. Energy variation study

The percentage depth dose (PDD) ratio of 20 cm to 10 cm is measured weekly with two Exradin A1SL ion chambers (Standard Imaging Inc., Middleton, WI) in Solid Water blocks and a static gantry. One of the two chambers is used to measure dose at various depths, while the other is used as a reference to monitor any output fluctuations. The PDD ratio is used as a metric for the beam energy with which TQA quantities are compared. For the unit that has undergone two energy adjustments, Pearson's correlation coefficient r is computed between each of the TQA quantities and the PDD ratio.

We then determine how sensitively the TQA quantities can track changes in the PDD ratio. TQA quantities with |r|>0.85 are plotted against the PDD ratios and are least‐square fitted. We define another quantity $\sigma_{\text{PDD}}$ by the following relation:

(1) $$\sigma_{PDD}=\frac{\sigma_{TQA}}{m}$$

where $\sigma_{TQA}$ is the standard deviation of the residuals from the fit of the TQA quantity, and *m* is the slope of the line of best fit. Note that for an energy fluctuation resulting in a PDD ratio variation less than $\sigma_{\text{PDD}}$, one cannot observe a corresponding statistically significant change in the TQA quantity. Therefore, by comparing the $\sigma_{\text{PDD}}$ for each TQA quantity, one can have a quantitative comparison of the sensitivities with which the TQA quantities can track energy fluctuations.

### B. Magnetron status monitoring

The second goal of this study is to find the TQA quantity or data trend most indicative of a magnetron failure. Before a magnetron fails, some instability is expected to arise in the output. The instability is investigated both quantitatively and qualitatively in this study. For the former, we look for a quantitative measure of the dose stability. Three quantities in Basic Dosimetry help track pulse stability: Pulse‐by‐Pulse Dose1, Pulse‐by‐Pulse Cone Variation, and Pulse‐by‐Pulse Exit Detector Average. These pulse‐by‐pulse quantities measure respectively the fluctuations of the dose output, cone shape (the lateral profile of the beam), and detector signal between successive pulses. Pulse‐by‐Pulse Dose1 in particular tracks the dose output variation between pulses, and hence will most likely detect anomalies in magnetron output. In this analysis, the one‐week averages of Pulse‐by‐Pulse Dose1 before and after each of the nine magnetron failures studied are compared using a Welch's t‐test. For the qualitative aspect of the study, the raw dose output profiles are surveyed in relation to magnetron failures.

## III. RESULTS

### A. Energy variation study

**Figure 1:**
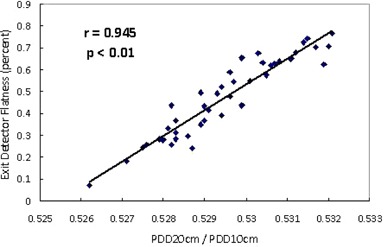
Exit Detector Flatness (Basic Dosimetry) versus PDD ratio plot. Table 1. Sensitivity of TQA quantity in relation to changes in PDD ratio.

Among the TQA quantities in the three modules, three are found to bear a strong correlation with the PDD ratio (p<0.01): Exit Detector Flatness in Basic Dosimetry (r=0.945, Fig. 1), and the two Energy Differences from Step‐Wedge Static (r=0.942) and Step‐Wedge Helical (r=0.898). By definition, the three quantities are defined to be zero at the time of commissioning. We then calculate $\sigma_{\text{PDD}}$ to compare the sensitivities with which the three TQA quantities can track changes in the PDD ratio. Table 1 summarizes the values of $\sigma_{\text{PDD}}$ for the three TQA quantities.

**Table 1:** Sensitivity of TQA quantity in relation to changes in PDD ratio

*TQA Quantity*	$\sigma_{PDD}(\times 10^{-4})$
Exit Detector Flatness (Basic Dosimetry)	5.1
Energy Difference (Step‐Wedge Static)	5.4
Energy Difference (Step‐Wedge Helical)	7.1

### B. Magnetron status monitoring

**Figure 2:**
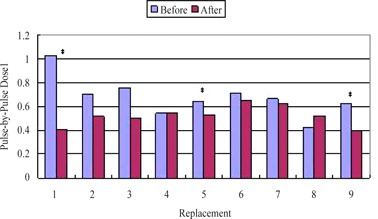
Average of Pulse‐by‐Pulse Dosel before and after each magnetron replacement. Asterisk denotes a statistically significant reduction in Pulse‐by‐Pulse Dosel after the replacement (p<0.05).

As shown in Fig. 2, generally Pulse‐by‐Pulse Dose1 reduces after each magnetron replacement; however, the reduction is only statistically significant (p<0.05) in three of the nine magnetron replacements.

**Figure 3:**
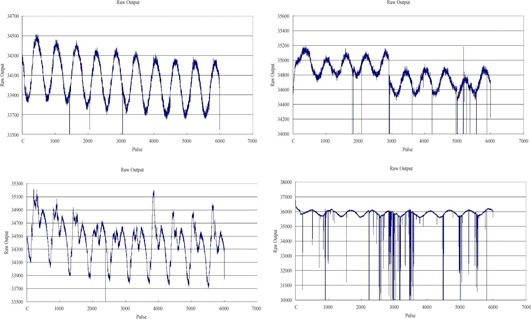
Examples of normal raw output (top left) and abnormal raw output (top right, bottom left, bottom right)

For a qualitative study of pulse‐by‐pulse stability, the raw outputs automatically plotted in Basic Dosimetry are visually inspected. The top left plot of Fig. 3 is an example of a normal raw output, where it is regular, periodic, and free from discontinuities in the waveforms. Occasional dropped pulses can be observed, but the frequency is not severe. Instabilities in the output can manifest in frequent dropped pulses, discontinuities in waveform or surges observed in the pattern, as shown in the other three plots of Fig. 3. Among the nine cases of magnetron failure studied, eight are found to be preceded by irregularities in the raw output. However, two of them occur on the day of the magnetron replacement and may not be useful in scheduling a magnetron replacement in advance. On the other hand, if the waveform displays no irregularities, a magnetron replacement is not needed for the following week 89% of the time (607 out of 682 times). The specificity, not sensitivity, of a visual inspection for abnormal waveforms may be of particular interest to users whose machines are not equipped with a dose servo. Such machines are especially vulnerable to magnetron instability.

## IV. DISCUSSION & CONCLUSION

### A. Energy variation study

For a beam with a higher energy, the cone tends to be more forward‐peaked. This in turn will increase the Exit Detector Flatness value, which is the root mean square of the normalized ratio between the measured and the reference cone profiles.^(3)^ The Energy Differences in the two step‐wedge modules alternatively utilize the attenuation profile of the aluminum step wedge to calculate the energy of the beam.^4^, ^5^ In our study, Exit Detector Flatness is found to be most sensitive in detecting beam energy drifts. However, it does not indicate whether the energy is too high or too low; it merely indicates that the cone shape has changed. Energy adjustments still rely on an external measurement with ion chamber. Nevertheless, Exit Detector Flatness may still be valuable in monitoring beam energy in addition to PDD ratio. First, the relatively high frequency by which Basic Dosimetry is run is advantageous in detecting any sudden changes in the energy. Second, Exit Detector Flatness is computed by taking into account the entire cone shape, whereas PDD ratio is measured only along the center of the beam. Variations in the cone shape can have an impact in dose distributions.

### B. Magnetron status monitoring

A reduction in the Pulse‐by‐Pulse Dose1 indicates that there is less pulse‐to‐pulse fluctuation in the dose. This is expected after a magnetron replacement when the output stability is restored. However, only some instances of magnetron replacement are found to reduce Pulse‐by‐Pulse Dose1 in a statistically significant way. In the case of a visual inspection of the raw output waveforms, abnormalities can be due to excessive magnetron arcing. When excessive arcing occurs, reconditioning or even replacing the magnetron may be necessary.

## ACKNOWLEDGMENTS

We would like to thank Wai Leung and Jackie Kwok from the Biomedical Engineering Department for their indispensable input.
